# Salivary Detection of Zika Virus Infection Using ATR-FTIR Spectroscopy Coupled with Machine Learning Algorithms and Univariate Analysis: A Proof-of-Concept Animal Study

**DOI:** 10.3390/diagnostics13081443

**Published:** 2023-04-17

**Authors:** Stephanie Wutke Oliveira, Leia Cardoso-Sousa, Renata Pereira Georjutti, Jacqueline Farinha Shimizu, Suely Silva, Douglas Carvalho Caixeta, Marco Guevara-Vega, Thúlio Marquez Cunha, Murillo Guimarães Carneiro, Luiz Ricardo Goulart, Ana Carolina Gomes Jardim, Robinson Sabino-Silva

**Affiliations:** 1 Innovation Center in Salivary Diagnostic and Nanobiotechnology, Department of Physiology, Institute of Biomedical Sciences, Federal University of Uberlandia, Uberlandia 38408-100, Brazil; 2 College of Dentistry, University Center of Triangle (UNITRI), Uberlandia 38411-869, Brazil; 3 Laboratory of Antiviral Research, Institute of Biomedical Science, Federal University of Uberlandia, Uberlandia 38408-100, Brazil; 4 Institute of Biosciences, Humanities and Exact Sciences, São Paulo State University, São José do Rio Preto 15054-000, Brazil; 5 School of Medicine, Federal University of Uberlandia (UFU), Uberlandia 38408-100, Brazil; 6 Faculty of Computing, Federal University of Uberlandia (UFU), Uberlandia 38400-902, Brazil; 7 Institute of Biotechnology, Federal University of Uberlandia, Uberlandia 38408-100, Brazil

**Keywords:** Zika virus, saliva, diagnosis, mice, ATR-FTIR

## Abstract

Zika virus (ZIKV) diagnosis is currently performed through an invasive, painful, and costly procedure using molecular biology. Consequently, the search for a non-invasive, more cost-effective, reagent-free, and sustainable method for ZIKV diagnosis is of great relevance. It is critical to prepare a global strategy for the next ZIKV outbreak given its devastating consequences, particularly in pregnant women. Attenuated total reflection–Fourier transform infrared (ATR-FTIR) spectroscopy has been used to discriminate systemic diseases using saliva; however, the salivary diagnostic application in viral diseases is unknown. To test this hypothesis, we intradermally challenged interferon-gamma gene knockout C57/BL6 mice with ZIKV (50 µL,105 FFU, *n* = 7) or vehicle (50 µL, *n* = 8). Saliva samples were collected on day three (due to the peak of viremia) and the spleen was also harvested. Changes in the salivary spectral profile were analyzed by Student’s *t* test (*p* < 0.05), multivariate analysis, and the diagnostic capacity by ROC curve. ZIKV infection was confirmed by real-time PCR of the spleen sample. The infrared spectroscopy coupled with univariate analysis suggested the vibrational mode at 1547 cm^−1^ as a potential candidate to discriminate ZIKV and control salivary samples. Three PCs explained 93.2% of the cumulative variance in PCA analysis and the spectrochemical analysis with LDA achieved an accuracy of 93.3%, with a specificity of 87.5% and sensitivity of 100%. The LDA-SVM analysis showed 100% discrimination between both classes. Our results suggest that ATR-FTIR applied to saliva might have high accuracy in ZIKV diagnosis with potential as a non-invasive and cost-effective diagnostic tool.

## 1. Introduction

Zika virus (ZIKV) is a flavivirus in the family Flaviviridae of RNA viruses. ZIKV is a disease primarily transmitted to humans by infected mosquitoes [1] and shares some clinical signs and symptoms, such as headaches, fever, and body aches, with dengue and chikungunya fever. Robust pathophysiological evidence supports a relationship between ZIKV infection in pregnancy and fetal brain adverse outcomes [2], such as fetal microcephaly in Zika-virus-infected pregnant women [3]. Furthermore, additional neurological diseases have been related to ZIKV, such as Guillain–Barré syndrome [4,5]. The last outbreak in Brazil spread throughout the Americas and was considered a public health emergency of international concern by the World Health Organization (WHO) [6]. Various infected Aedes mosquito species, including A. aegypti and A. albopictus, are vectors of ZIKV due to the presence of infectious saliva. Non-mosquito transmission of ZIKV from an infected mother to a fetus in pregnancy (although the ZIKV transmission risk has not been determined), sexual transmission [7,8], and blood transfusion have been reported [9].

The clinical routine of ZIKV infection diagnosis is based on viral nucleic acid using RT-PCR within 7 days after the onset of symptoms or with detection of specific IgM antibodies against ZIKV using immunoassays. The latter is more efficient within the first week of clinical infection and can remain detectable [1]. ZIKV has been identified based on viral detection by real-time polymerase chain reaction (RT-PCR) in breast milk [10], blood, semen, urine [11], and saliva [12] in the acute phase of infected patients. Furthermore, it is important to point out that infective ZIKV isolation from saliva has also occurred in humans [13].

Salivary biomarkers have been considered an attractive alternative for the early detection of systemic diseases [14]. Saliva is a dynamic biofluid composed of ~98% water and 2% other organic and inorganic components, such as electrolytes, mucus, enzymes, proteins/peptides, nucleic acids, and hundreds of microorganism species, such as viruses [15]. Saliva collection is simple, fast, safe, convenient to store, non-invasive, and, compared to blood, is painless and requires less handling during diagnostic proceedings [16]. There is great potential for ZIKV detection in saliva during the acute phase of infection in patients and for detection of ZIKV antibodies, such as IgM, for several months after [12]. Salivary diagnosis of ZIKV infection using alternative diagnostic platforms with advantages compared to the RT-PCR should be considered.

Infrared (IR) spectroscopy is a powerful quantitative and qualitative analytical platform to characterize biological components in biofluids. The attenuated total reflection–Fourier transform infrared (ATR-FTIR) spectroscopy is a rapid, sustainable, reagent-free, and high-sensitive platform that detects molecular bonds by atomic displacement [17]. ATR-FTIR requires a small volume of sample for each analysis with simple preparation and allows automated and high-throughput analyses [18,19]. As biochemical changes are frequently described in emerging diseases, the use of ATR-FTIR can reveal these differences at a molecular level and could serve as a novel screening and/or diagnostic tool [17,20,21,22,23,24].

Saliva contains thousands of proteins, salivary peptides, mRNA, extracellular RNAs, microRNAs, metabolites, lipids, carbohydrates, and hundreds of microorganism species, such as viruses, that could act as unique sources of biomarkers [25]. ZIKV infection is capable of inducing the presence of ZIKV in saliva [12] and of changing other unique salivary components in response to ZIKV pathogenesis [26]. Analyzing biofluids in ATR-FTIR platforms can detect specific molecular fingerprints from proteins, peptides, RNAs, lipids, and carbohydrates by their unique chemical bonds [21,27]. As such, differentially expressed salivary components derived from ZIKV infection could be detected by the ATR-FTIR platform. In this study, we tested the hypothesis that unique spectral salivary biomarkers can be differentially expressed in the saliva of ZIKV-infected mice, and we explored how potential vibrational modes can be used as salivary biomarkers for ZIKV detection. Thus, our study aimed to identify a salivary infrared profile to detect ZIKV signatures that are suitable for screening or for performing a diagnosis of this emerging disease.

## 2. Materials and Methods

### 2.1. Animals

All experimental procedures were approved by the Ethics Committee for Animal Research of the Federal University of Uberlandia (UFU) (license #CEUA-UFU #071/2017). The procedures were carried out under the International Guiding Principles for Biomedical Research Involving Animals of the International Council for Laboratory Animal Science (ICLAS), countersigned by the Brazilian National Council for the Control of Animal Experimentation (CONCEA). The ethical design of this study followed the 3R principles of animal experimentation: reduction, replacement, and refinement.

### 2.2. Induction of ZIKV Infection, Saliva, and Sample Collection

**Figure 1:**
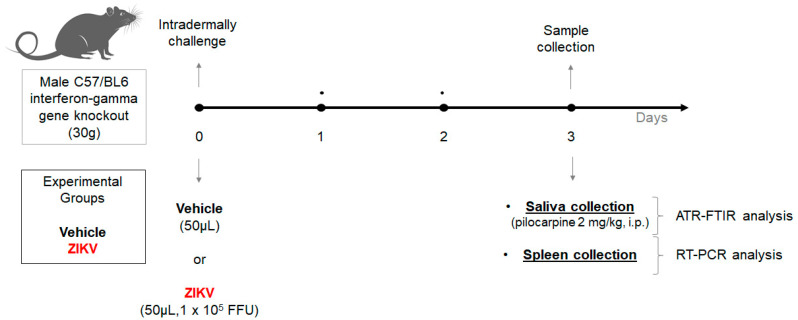
Experimental workflow of Zika virus infection in animal model using male C57/BL6 interferon-gamma gene knockout for salivary analysis using ATR-FTIR platform.

Two-month-old interferon-gamma gene knockout C57/BL6 male mice (30 g) from the Rodent Animal Facility Network (REBIR/PROPP/UFU) were divided into vehicle (*n* = 8) and ZIKV (*n* = 7) groups. The animals were maintained under standard conditions (22 ± 1 °C, 60% ± 5% humidity, and 12-h light/dark cycles, light on at 7 AM) and were allowed free access to standard diet and water in a rodent facility. ZIKV infection was induced by an intradermal challenge with ZIKV (50 µL, 1 × 10^5^ FFU) and control mice received vehicle (50 µL). At day 3, due to the peak of viremia, the animals were anesthetized with 12 mg/kg of xylazine and 80 mg/kg of ketamine [28], and subsequently, both vehicle and ZIKV mice received pilocarpine (2 mg/kg, i.p.) to stimulate salivary secretion. The saliva was collected for 5 min and the samples were frozen at −80 °C. Furthermore, the spleen was also removed (Figure 1).

After the intradermal challenge with vehicle or ZIKV, we performed a careful analysis in animals to perform a humane endpoint with specific criteria: (1) ruffled fur and ocular discharge; and (2) in cases of ataxia, tremor, and cyanosis [29]. However, it was not necessary to perform euthanasia in this set of vehicle and ZIKV animals.

### 2.3. ZIKV Infection Confirmation Using Reverse Transcription-Quantitative Polymerase Chain Reaction (RT-qPCR)

ZIKV mRNA detection was analyzed by real-time reverse transcription polymerase chain reaction (RT-PCR) in the spleen.

ZIKV mRNA detection was analyzed by reverse transcription-quantitative polymerase chain reaction (RT-qPCR) in the spleen. Viral RNA was extracted from spleen samples using the Direct-zolTM RNA Miniprep Plus Kit (Zymo Research) following the manufacturer’s protocol. Furthermore, RNA was converted to cDNA using the High-Capacity cDNA Reverse Transcription Kit (Applied Biosystem, Waltham, MA, USA). The RT-qPCR assay was prepared following SYBR Green JumpStart Taq ReadyMix (SIGMA) protocol using ZIKV-specific primers (5 pmol). Forward: 5′TTGGTCATGA TACTGCTGATTGC3′and Reverse: 5′CCTTCCACAAAGTCCCTATTGC3′ [30]. The RT-qPCR was carried out in an Applied Biosystem 7300 system for 40 cycles at 95 °C for 15 s and 60 °C for 1 min, and 1 cycle at 95 °C for 15 s, 60 °C for 1 min, 95 °C for 15 s and 60 °C for 15 s. Each sample was analyzed in triplicates. Positive and negative template control was also included in all experiments.

### 2.4. Chemical Profile in Stimulated Saliva by ATR-FTIR Spectroscopy

Salivary spectra were recorded in the transmission mode (OPUS v.6.5 software, Bruker) using ATR-FTIR spectrophotometer Vertex 70 (Bruker Optics) using a micro-attenuated total reflectance (ATR) component. The Eppendorf devices with saliva were inserted in a vortex for 5 min to homogenate the sample, and 2 μL were collected and directly dried on ATR-crystal with a dry dentistry airflow for 5 min for infrared spectra recording. Spectra were acquired with 4 cm^−1^ spectral resolution and 32 scans per sample, from 400 to 4000 cm^−1^. Two replicates per sample were studied and the mean was used for each sample [21,23,31].

### 2.5. Spectra Data Procedures

The original spectra were analyzed in the OPUS software environment. Each raw spectrum was truncated in the biofingerprint region (1800–900 cm^−1^) for univariate analysis, followed by a normalization by vector and a subsequent baseline correction. The second differentiation spectra from the raw spectra were carried out using the Savitzky–Golay method in Origin 9.1 software. The parameters were set as 2 for polynomial order and 20 for points of the window. The second derivative provides some valleys (negative peaks) based on bands from the original absorption spectrum. Therefore, we calculated the level and ROC curve data based on the height of valleys in the second derivative [24].

The 1800–900 cm^−1^ region of the original spectrum was used as input data for the multivariate analysis using principal component analysis (PCA). PCA was applied to the initial exploratory analysis of the spectral data. In this analysis, the PC was assembled with scores (deviation in sample orientation) and loadings (variance in the wavenumber direction). The similarity level between samples was assessed by the scores, and the loadings were displayed by the weight of each wavenumber towards the score pattern [24]. All pre-processing and spectral analysis steps were performed with Origin Pro 9.1 (OriginLab Corporation, Northampton, MA, USA) [32].

For the machine learning algorithms, the spectral data were pre-processed considering an attribute selection method, which is responsible for truncating each spectrum associated with the lipidic region (3050–2800 cm^−1^) and with the biofingerprint region (1800–900 cm^−1^). The supervised models based on linear discriminant analysis (LDA) obtained an adequate classification model to systematically distinguish salivary samples of ZIKV from controls (vehicle). Based on the generalization of Fisher’s linear discriminant, LDA works by projecting the original data onto a lower-dimensional space that maximizes class separation and reduces computational costs [21,33]. Support vector machine (SVM) has been contributed in ATR-FTIR spectroscopy with improved results compared with other learning algorithms. First, SVM projects the input data into a high-dimensional space, and then it fits hyperplanes to separate the data classes [33].

### 2.6. Statistical Analysis

The data were analyzed using Student’s *t* test. For all spectral biomarker candidates, we constructed the Receiver Operating Characteristic (ROC) curve. The Kolmogorov–Smirnov test was applied to assess the normality of variables. All analyses were performed using GraphPad Prism. Values of *p* < 0.05 were considered significant and the results were expressed as mean ± S.D.

## 3. Results

**Figure 2:**
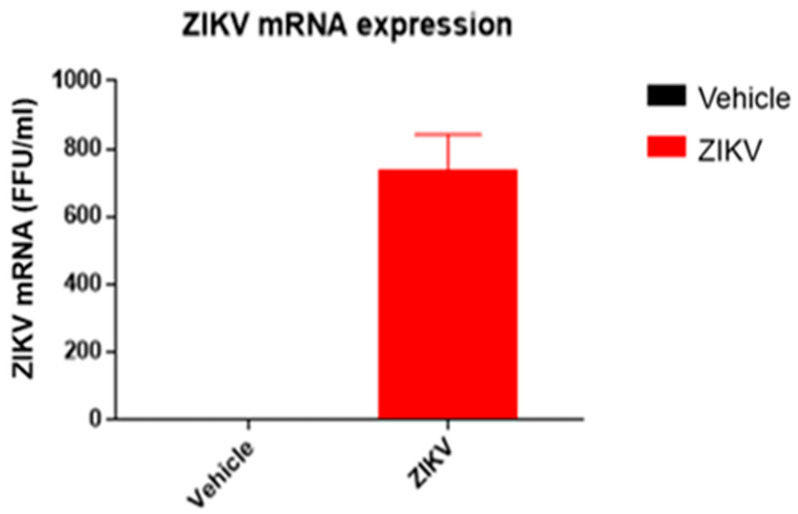
ZIKV mRNA was detected in spleen samples of ZIKV mice, confirming viral infection. ZIKV RNA measured by RT-qPCR in spleen samples of vehicle and ZIKV mice. The RT-qPCR assay was analyzed in triplicates for all samples.

The body weight of ZIKV-infected mice (ZIKV: 25.7 ± 0.9 g) was similar (*p* > 0.05) to vehicle mice. ZIKV RNA replication was present only in the spleens of ZIKV mice (ZIKV: 730 ± 155 FFU/mL; Vehicle: 0 FFU/mL (Figure 2)).

### 3.1. Univariate Analyses of the ATR-FTIR Diagnostic Model

**Figure 3:**
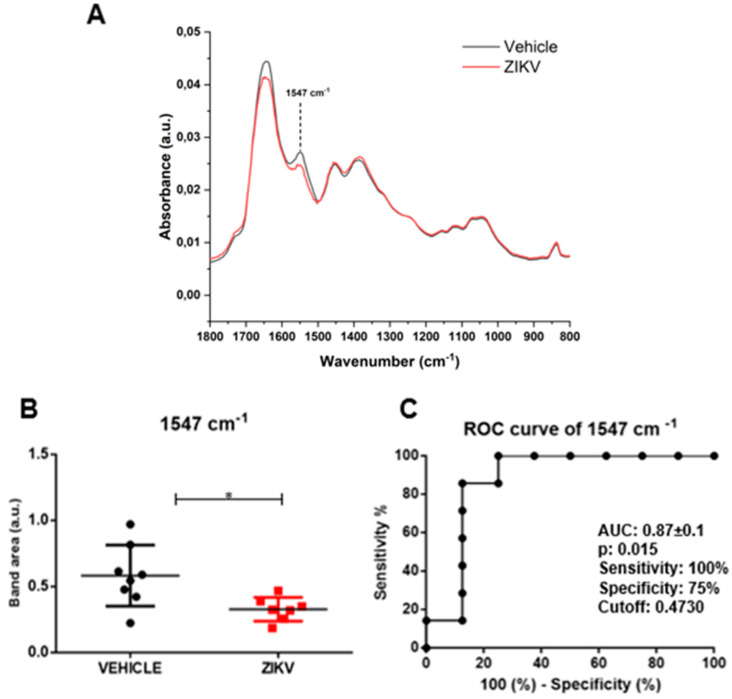
The band area at 1547 cm^−1^ was reduced in ZIKV mice compared to vehicle mice, with significant changes in ROC curve analysis. Representative average ATR-FTIR original spectra normalized by norm (1800–800 cm^−1^) in saliva of vehicle mice and ZIKV mice (**A**). Band area at 1547 cm^−1^ (**B**). ROC curve analysis of band area at 1547 cm^−1^ in original spectra (**C**). Results are mean ± SEM of 8 vehicle mice and 7 ZIKV mice; * *p*  <  0.05 vs. vehicle.

Raw data of each sample and representative average with a standard deviation of ATR-FTIR spectra (4000–400 cm^−1^) in saliva of vehicle and ZIKV mice are depicted in Supporting Figure S1. The mean infrared original spectrum normalized by norm of saliva in the fingerprint region (1800–800 cm^−1^) collected from the vehicle and ZIKV mice indicates a superposition of salivary components, such as proteins, lipids, DNA/RNA, and carbohydrates (Figure 3). These pre-processed salivary spectra of vehicle and ZIKV mice were further evaluated by univariate analysis (band area of original spectra and height of valleys of the second derivative spectra) and multivariate analysis (principal component analysis (PCA) and linear discriminant analysis (LDA)). The infrared spectral band areas were analyzed in the saliva of vehicle and ZIKV mice; however, only the band area at 1547 cm^−1^ (1577 cm^−1^–1500 cm^−1^) was changed in ZIKV mice compared to vehicle mice. The original spectra band area at 1547 cm^−1^ was reduced (*p* < 0.05) in ZIKV mice compared to vehicle mice (Figure 3A). The ROC curve analysis was applied in the 1547 cm^−1^ vibrational mode to determine the sensitivity, specificity, and accuracy of discrimination between ZIKV and vehicle mice. The selected cutoff value was 0.47 a.u. to the vibrational mode at 1547 cm^−1^ (Figure 3B); this indicates sensitivity and specificity of 100% and 75%, respectively. Furthermore, the area under the curve (AUC) of this vibrational mode was 0.87 (*p* < 0.05; Figure 3C).

**Figure 4:**
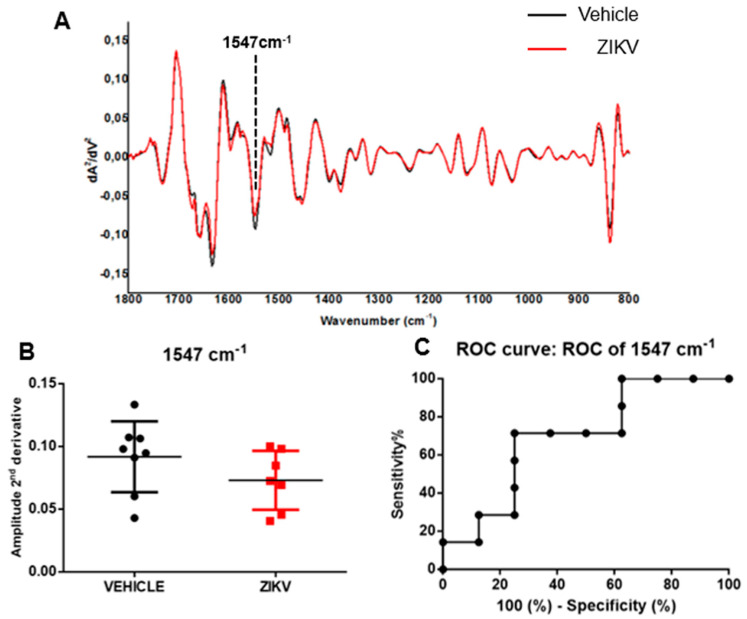
The amplitude of second derivative infrared spectrum at 1547 cm^−1^ vibrational mode was significant in ROC curve analysis comparing ZIKV mice with vehicle mice. Representative average ATR-FTIR second derivative spectra (1800–800 cm^−1^) in saliva of vehicle mice and ZIKV mice (**A**). The amplitude of second derivative at 1547 cm^−1^ (**B**). ROC curve analysis of 1547 cm^−1^ in the second derivative spectra (**C**). Results are mean ± SEM of 8 vehicle mice and 7 ZIKV mice.

The average of the second derivative infrared spectrum of saliva in vehicle mice and ZIKV mice is represented in Figure 4A. The height of valleys (amplitude) in saliva samples was similar (*p* > 0.05) in several vibrational modes evaluated in the second derivative spectra in vehicle mice and ZIKV mice. We identified the height of valley amplitude at 1547 cm^−1^ in accordance with the previous analysis in the original infrared spectra (Figure 4B). The ROC curve of 1547 cm^−1^ vibrational mode presented 71% sensitivity and 75% specificity for ZIKV discrimination and the AUC of this vibrational mode was 0.69 (*p* > 0.05; Figure 4C).

### 3.2. Multivariate Analyses of ATR-FTIR Diagnostic Model

**Figure 5:**
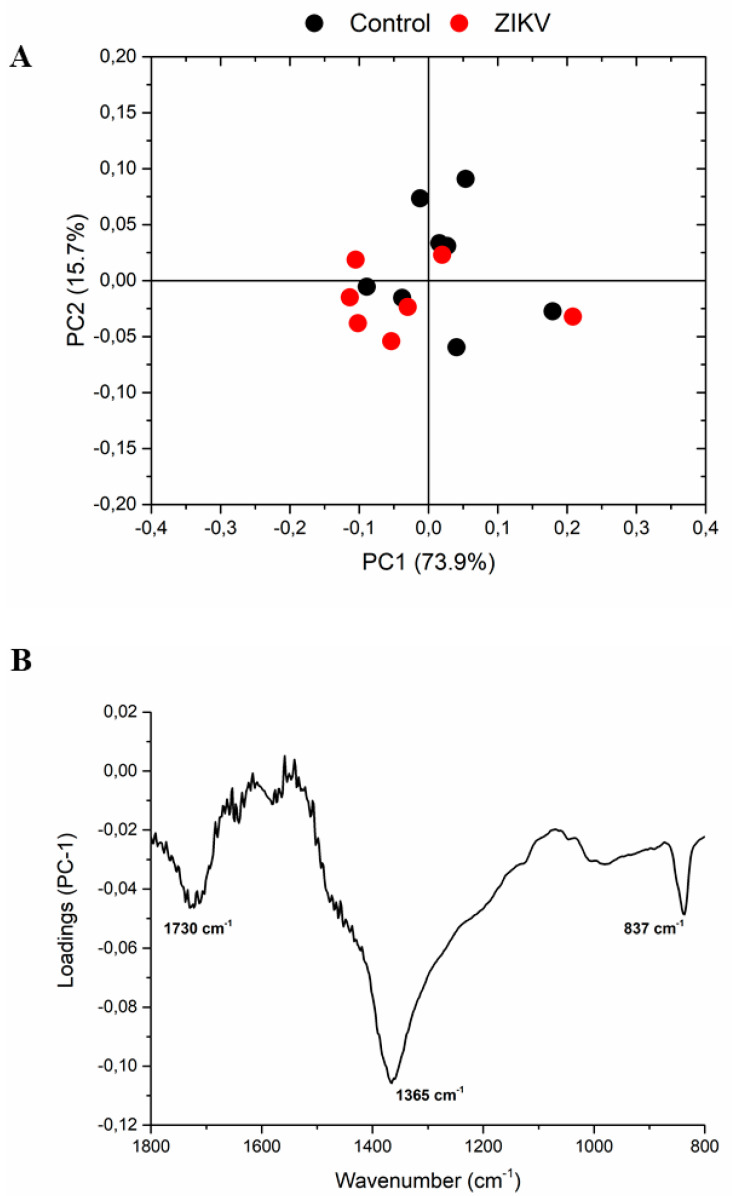
The principal component analysis (PCA) displayed significant changes in salivary spectra of vehicle mice and ZIKV mice. Principal component analysis score plot to PC1 vs. PC2 (**A**) and PCA loadings to PC1, PC2, and PC3 (**B**). The vibrational modes at 1730 cm^−1^, 1365 cm^−1^, and 837 cm^−1^ were used with high score to discriminate both groups.

The infrared fingerprint region (1800–800 cm^−1^) from the vehicle mice and ZIKV mice was subjected to an initial exploratory analysis to detect potential infrared spectral changes in both groups. The principal component analysis (PCA) displayed significative differences in salivary spectra of vehicle and ZIKV mice. Three principal components (PCs) named PC1, PC2, and PC3 explained 93.2% of cumulative variance. Both PC1 (73.9% explained variance) and PC2 (15.7% explained variance) are represented in the score plot (89.6% explained variance in Figure 5A). The PCA loadings to PC1, PC2, and PC3 are represented in Figure 5B. We highlighted the higher absolute coefficients represented by these subsequent vibrational modes: 1656, 1585, and 1417 cm^−1^ in PC3; 1639 and 1538 cm^−1^ in PC2; and 1730, 1365, and 837 cm^−1^ in PC1.

**Figure 6:**
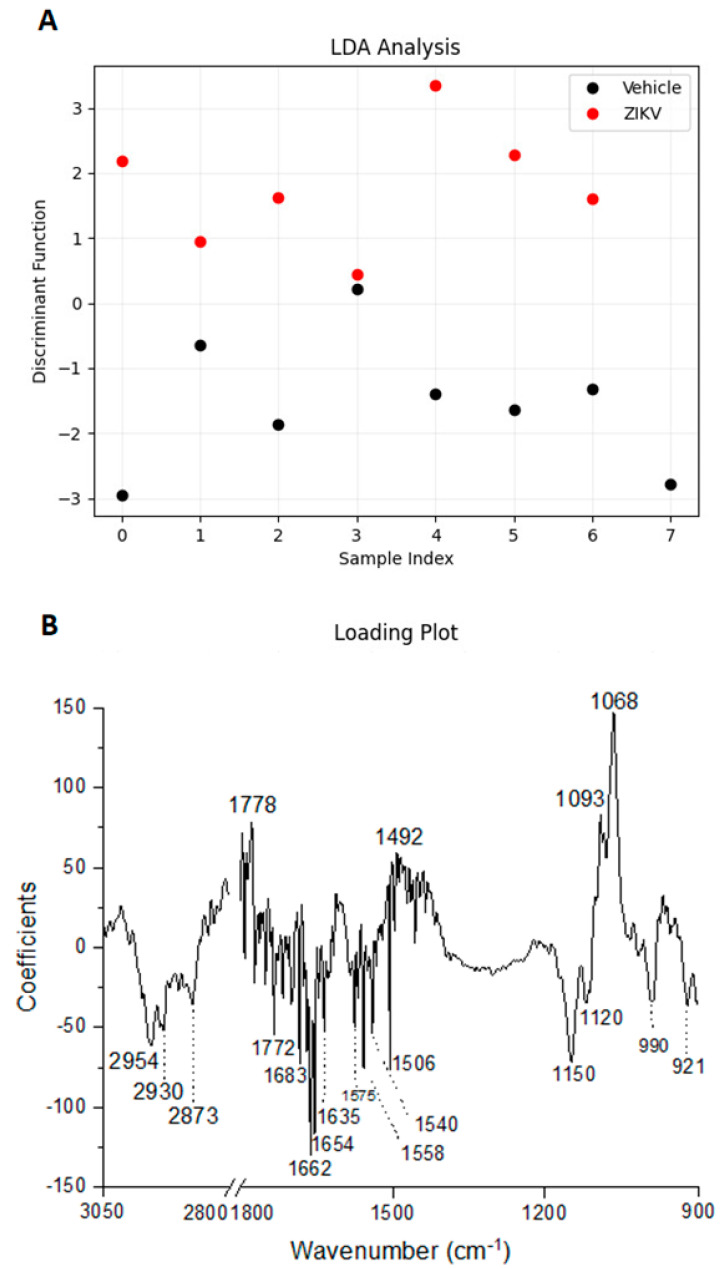

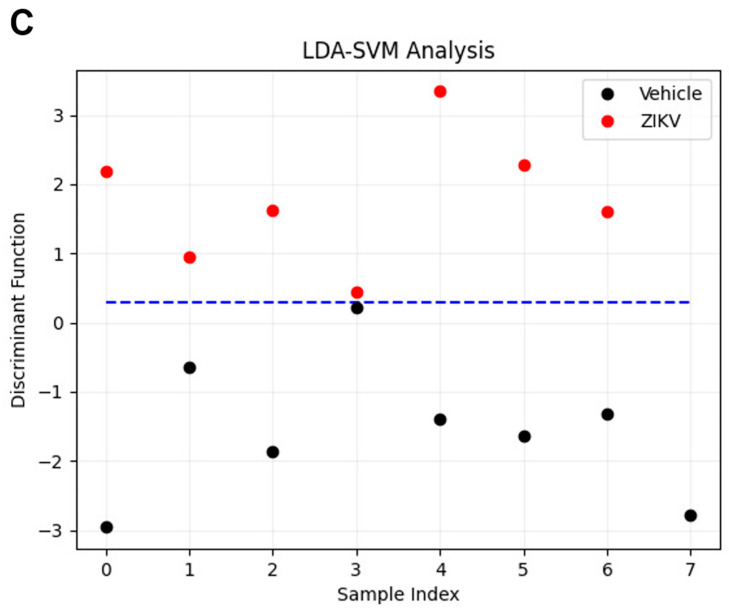
Learning machine algorithms were capable of discriminating changes in salivary spectra of vehicle mice and ZIKV mice with high accuracy. Linear discriminant analysis (LDA) discriminant function plot (**A**), LDA loading plot (**B**), and linear support vector machine (SVM) model over the LDA projected data. The blue dotted line indicates the decision boundary learned by the SVM algorithm for the discrimination of the ZIKV and Vehicle samples (**C**).

Considering the discriminant function presented in the *Y*-axis, the vehicle mice and ZIKV mice salivary samples were distributed in Figure 6A. All seven salivary samples from ZIKV mice were distributed above zero, and seven of eight salivary samples from vehicle mice were distributed below zero. Despite the projected data, LDA also fits a predictive model to each class based on Gaussian density. Such a model misclassified only the salivary sample from vehicle mice that was positioned above zero. Therefore, the accuracy obtained for the classification of the dataset was 93.3% (14/15), with a specificity of 87.5% (7/8) and sensitivity of 100% (7/7). The loading plot with spectral wavenumbers responsible for discrimination between control and ZIKV is represented in Figure 6B. In addition, we also trained a linear SVM model over the LDA projected data. In Figure 6C, such a learned model is represented by the dotted lines, which demonstrates 100% discrimination between both classes. In summary, the specificity, sensitivity, and accuracy of the LDA and SVM classifications were calculated as described in Table 1.

The tentative assignment for the main wavenumbers responsible for discrimination between non-infected controls and ZIKV-infected animals is presented in Table 2. These data suggest changes in the expression of lipids, phosphodiesters, carbohydrates, and, mainly, proteic components in saliva samples to discriminate non-infected controls and ZIKV-infected animals.

## 4. Discussion

The implementation of non-invasive and reagent-free platforms for active ZIKV diagnosis using saliva has a powerful impact on early diagnosis, which is pivotal to successful effective treatment. In this context, this study is a pioneer as a proof-of-concept study to evaluate the translational applicability of a photonic platform for screening or diagnosing ZIKV in saliva. The infrared spectroscopy coupled with univariate analysis suggested the vibrational mode at 1547 cm^−1^ as a potential candidate to discriminate ZIKV and control salivary samples. Three PCs explained 93.2% of cumulative variance in PCA analysis and the spectrochemical analysis with LDA achieved an accuracy of 93.3%, with a specificity of 87.5% and sensitivity of 100%. The ~2950 cm^−1^, ~1650 cm^−1^, and ~1070 cm^−1^ regions were responsible for reliability to discriminate control and ZIKV, which suggests that salivary detected alterations occur in lipid, protein, and carbohydrate components. Furthermore, the LDA-SVM analysis showed 100% discrimination between both control and ZIKV salivary samples. Altogether, these data indicate a great potential for ATR-FTIR analysis to discriminate ZIKV from controls using saliva.

As expected, the present ATR-FTIR profile of saliva from non-infected control rodents and healthy humans was capable of indicating protein, lipidic, carbohydrates, and nucleic acids components, as described previously [21,34,35,36]. Saliva samples increased the molecular detection rate of viral RNA during the acute phase of ZIKV compared to blood; however, the ZIKV RNA window detection was similar in both samples [12]. ZIKV peptides were detected in saliva during the convalescent phase when the ZIKV RNA was not detected in saliva [5]. Although clinical signs and symptoms of ZIKV infection include fever, headache, arthralgia, myalgia, and maculopapular rash, these characteristics cannot be used as an effective diagnostic tool. This is because other suspected arboviral diseases, such as dengue and chikungunya, present similar symptomatology, and approximately 80% of the ZIKV-infected population are likely asymptomatic [37].

Screening and diagnostic testing are critical to apply both personalized medicine and public health control measures [38]. The diagnosis of ZIKV infection currently relies on the detection of viral RNA via reverse transcription-quantitative polymerase chain reaction (RT-qPCR) or identifying an IgM serologic response. Given testing limitations, ZIKV diagnosis presents some limitations in the sensitivity of RT-PCR and antibody cross-reactivity (IgM) tests. Considering that the exact timing of ZIKV infection is frequently unknown, it is critical to select the appropriate diagnostic test with high sensitivity and specificity to detect the virus genome by RT-qPCR in acute response, or specific antibodies as a secondary response. In this context, this study detected changes in the salivary infrared spectra in the acute phase of ZIKV infection, suggesting that other vibrational modes can be activated in the secondary responses.

Our data support our hypothesis that infrared vibrational modes of saliva may discriminate ZIKV-infected mice from control mice. Here, we have identified a new salivary ATR-FTIR spectral biomarker for ZIKV screening using univariate analysis. The 1547 cm^−1^ salivary vibrational mode indicates that amide II could potentially be used as a salivary biomarker with acceptable accuracy. ROC curve analysis is a statistically valid method for biomarker performance evaluation [39] and, in this study, the ROC curve analysis showed high accuracy for the 1547 cm^−1^ vibrational mode in both band areas in the original spectra and the amplitude of second derivative analysis. The concordance of salivary secretion mechanism in animal models and humans suggests this salivary vibrational mode as a potential candidate to be applied also in the diagnosis of ZIKV in humans.

Unsupervised PCA analysis was applied to evaluate similarities and dissimilarities of salivary spectra in salivary samples from control mice and ZIKV mice. The salivary vibrational modes at 1730 cm^−1^, 1656 cm^−1^, 1639 cm^−1^, 1585 cm^−1^, 1538 cm^−1^, 1417 cm^−1^, 1365 cm^−1^, and 837 cm^−1^ in PC1, PC2, and PC3 were pivotal for explaining 93.2% of cumulative variance between control and ZIKV samples, suggesting changes in the C = O stretching, amide I, C = C stretching, amide II, and polysaccharides in saliva. Bearing in mind the very low concentration of ZIKV in saliva, these spectral changes can be attributed also to secondary alterations in salivary components promoted by ZIKV infection.

Amide II is mainly associated with the bending vibration of the N–H bond and it is used to investigate the secondary structure of proteins. Furthermore, salivary peptide sequences with amide II have presented potential diagnostic applications in humans [40,41]. The presence of amide II could be attributed to the presence of nitrite (NO_2_^-^) compounds. Oral bacteria are pivotal to reducing nitrate (NO_3_^-^) in NO_2_^-^ [42]. In this context, we can expect changes in the oral microbiota of ZIKV-infected mice to parallel changes in gut microbiota modulation promoted by ZIKV infection [43].

Considering the obstacles to clinical applications, we also applied an additional supervised approach to evaluate the pattern classification in control and ZIKV salivary samples. The accuracy obtained for the pattern classification was 93.3%, with a specificity of 87.5% and sensitivity of 100%. The ~2950 cm^−1^, 1650 cm^−1^, and 1070 cm^−1^ regions were mainly responsible for discriminate control and ZIKV, which suggest that salivary detected alterations occur in lipid, protein, and carbohydrate/saccharides components. ZIKV is a single-stranded RNA virus that expresses E protein, M protein, and glycoproteins enveloped in a lipid membrane [44,45]. Although proteins, lipids, and glycoproteins are present in the ZIKV structure, the expected very low concentration of ZIKV in saliva suggests that these differences in salivary infrared spectra can also be attributed to changes promoted by ZIKV infection in the salivary glands or oral mucosa cells. Furthermore, the LDA-SVM analysis showed 100% discrimination between both control and ZIKV salivary samples. Table 2 suggests changes in the expression of 20 vibrational modes related to lipids, phosphodiesters, carbohydrates, and, mainly, proteic components on saliva samples to discriminate non-infected controls from ZIKV-infected animals.

In order to perform conventional diagnosis, blood tests have been required with significant clinical costs. Salivary ATR-FTIR diagnosis or screening could be a convenient alternative due to the potential for high-throughput screening and cost-effectiveness for characterization of spectrochemical signature (proteins, lipids, nucleic acids, and carbohydrates) of biofluid rather than focusing on a single specific protein as a biomarker [17].

To our knowledge, this is the first exploratory study using the ATR-FTIR platform to identify potential salivary biomarkers for diagnosing or screening ZIKV in an animal model. Therefore, to determine the clinical applicability of this green technology, further studies should be performed to validate the suggested spectral biomarkers in human saliva. Furthermore, it is imperative to compare results with salivary samples from other flaviviruses to find a unique spectral fingerprint that can discriminate ZIKV from other virus diseases, especially dengue and chikungunya infections. In future studies using human samples, we can compare the discrimination rate of this biophotonic platform with other arbovirus infections. The pioneering development of a fast, reagent-free, and non-invasive approach for the detection of ZIKV infection described here was based on a unique analysis of spectral salivary components that could be of great use to public health. Although we need further confirmation in human samples to determine that our ZIKV biophotonic platform is suitable to detect this viral infection, the present results suggest that our pioneering approach to detect ZIKV infection presents significant potential to discriminate using saliva samples of healthy subjects and ZIKV patients. Considering that specific diseases produce unique changes in infrared salivary profile, we can assume that our learning machine algorithms and multivariate chemometric analysis are suitable to discriminate between ZIKV infection and other viral infections.

It is also important to emphasize that ATR-FTIR has been used for biofluids analysis, allowing same-day detection and opening new opportunities for monitoring and diagnosing a range of diseases [21,22,23,46]. These salivary photonic-based diagnostics should be tested in large sample cohorts in patients and to open the possibility of point-of-care assays by portable infrared spectroscopic devices. Furthermore, it is noteworthy that ZIKV diagnosis remains imprecise, has a high cost, and that there is no gold-standard test [47]. In this context, the employment of a label-free ATR-FTIR has robust potential to reduce costs in ZIKV diagnostic. The prospect of identifying spectral biomarkers in saliva opens new opportunities, and we believe that these salivary ATR-FTIR-based diagnostics could be used in the future to diagnose the ZIKV disease using saliva samples rapidly and inexpensively, and even open the possibility for point-of-care assays by portable ATR-FTIR devices.

## 5. Conclusions

This proof-of-concept study demonstrated the potential of salivary infrared signatures to diagnose ZIKV with very high accuracy using a reagent-free ATR-FTIR platform. The infrared spectroscopy coupled with univariate analysis suggested the vibrational mode at 1547 cm^−1^ as a potential candidate to discriminate ZIKV and control salivary samples. Three PCs explained 93.2% of cumulative variance in PCA analysis, and the spectrochemical analysis with LDA achieved an accuracy of 93.3%, with a specificity of 87.5% and sensitivity of 100%. Furthermore, the LDA-SVM analysis showed 100% discrimination between both control and ZIKV salivary samples. A larger sample size should be tested in control, ZIKV, and other flaviviruses to test the potential of salivary infrared spectroscopy as a clinical diagnostic or screening test for ZIKV infection.

## Figures and Tables

**Table 1:** Distribution of the dataset for clustering classification in LDA and LDA-SVM analysis.

LDA						
**True Positive**	**True Negative**	**False Positive**	**False Negative**	**Specificity**	**Sensitivity**	**Accuracy**
7	7	1	0	87.5%	100%	93.3%
LDA-SVM						
**True Positive**	**True Negative**	**False Positive**	**False Negative**	**Specificity**	**Sensitivity**	**Accuracy**
7	8	0	0	100%	100%	100%

**Table 2:** The tentative assignments for loading plots with spectral wavenumbers responsible for discriminating between non-infected controls and ZIKV-infected animals using learning machine algorithms.

Vibrational Mode (cm^−1^)	Tentative Assignment
2954	Asymmetric stretching of CH3 of acyl chains (lipids)
2930	C-H stretching bands
2873	Symmetric stretching of CH3 of acyl chains (lipids)
1778	Lipids
1772	Lipids
1683	C = O Guanine deformation N-H in plane
1662	Amide I
1654	Alpha-helix conformation of Amide I
1635	Beta-sheet structure of amide I
1575	C = N adenine
1558	Amide II
1540	Amide III
1506	In-plane CH bending vibration from the phenyl rings
1492	Deformation C-H
1150	Phosphodiester stretching
1120	Mannose-6-phosphate
1093	Stretching PO2 2 symmetric
1068	Stretching C-O ribose
990	C-O ribose, C-C
921	Glycosidic linkage formation

## Data Availability

All relevant data were present in this manuscript.

## Supplementary Materials

The following supporting information can be downloaded at: https://www.mdpi.com/article/10.3390/diagnostics13081443/s1, Supporting Figure S1. Raw data of each sample (A) and representative average with a standard deviation (B) of ATR-FTIR spectra (4000–400 cm^−1^) in saliva of vehicle mice and ZIKV mice.
