# Supplementary material for: Salivary Detection of Zika Virus Infection Using ATR-FTIR Spectroscopy Coupled with Machine Learning Algorithms and Univariate Analysis: A Proof-of-Concept Animal Study
Source: Diagnostics (Basel). 2023 Apr 17;13(8):1443. doi: 10.3390/diagnostics13081443 (PMC10137856; doi:10.3390/diagnostics13081443)
Supplement: Supplementary file 1 [file diagnostics-13-01443-s001.zip › diagnostics-2238833-supplementary.pdf]

# Salivary Detection of Zika Virus Infection Using ATR-FTIR Spectroscopy Coupled with Machine Learning Algorithms and Univariate Analysis: A Proof-of-Concept Animal Study

**Stephanie Wutke Oliveira [1,†], Leia Cardoso-Sousa [1,†], Renata Pereira Georjutti [1,2], Jacqueline Farinha Shimizu [3,4], Suely Silva [3,4], Douglas Carvalho Caixeta [1], Marco Guevara-Vega [1], Thúlio Marquez Cunha [5], Murillo Guimarães Carneiro [6], Luiz Ricardo Goulart [7,‡], Ana Carolina Gomes Jardim [3,4] and Robinson Sabino-Silva [1,*]**

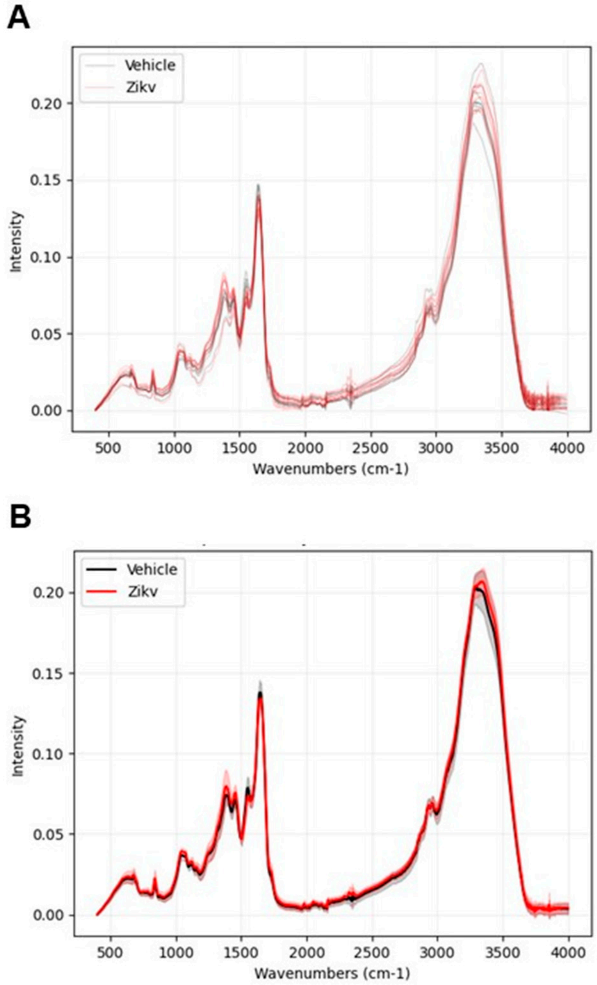


**Supporting Figure S1.** Raw data of each sample (**A**) and representative average with a standard deviation (**B**) of ATR-FTIR spectra (4000–400 cm$^{-1}$) in saliva of vehicle mice and ZIKV mice.
